# The application of the metaverse in surgical clinical teaching: transforming medical education through immersive approaches

**DOI:** 10.3389/fonc.2025.1626680

**Published:** 2025-08-13

**Authors:** Tao Yu, Zhichao Yang, Meng Zhang, Lin Yao, Xiaodan Sun

**Affiliations:** ^1^ Department of Gastrointestinal Surgery, Fuxin Central Hospital, Liaoning, China; ^2^ Department of Gastroenterology, Fuxin Central Hospital, Liaoning, China; ^3^ Department of Intensive Care Unit, Fuxin Central Hospital, Liaoning, China

**Keywords:** metaverse, surgical clinical teaching, medical education, virtual reality, interactive learning

## Abstract

With the rapid development of immersive technologies such as virtual reality (VR) and augmented reality (AR), the Metaverse is emerging as a transformative platform in medical education. This study examines the integration of the Metaverse into surgical clinical teaching, highlighting its potential to enhance educational outcomes, overcome traditional limitations, and foster global collaboration in medical training. Core features of the Metaverse offer medical students a safe virtual setting for practicing complex surgical procedures and honing clinical decision-making skills. However, significant challenges remain, including high technological costs, substantial equipment requirements, and the complexity of content development. Ethical concerns, particularly regarding data privacy and the psychological impact of immersive experiences, also require careful consideration. This paper calls for strategic planning, interdisciplinary collaboration, and ongoing research to fully realize the transformative potential of the Metaverse in surgical clinical education, ultimately improving the quality and effectiveness of medical training.

## 1 Introduction

With the rapid advancement of technology, immersive platforms such as virtual reality (VR) and augmented reality (AR) have begun to transform various sectors, including medical education (1, 2). Traditional surgical clinical teaching, however, faces numerous challenges. For example, many institutions experience significant disparities in resource allocation and limited access to advanced surgical facilities, which result in fewer hands-on opportunities for students. Specific cases have shown that students in resource-constrained settings often miss out on practicing complex procedures, leading to a gap between theoretical knowledge and practical proficiency (3, 4). Such limitations not only hinder skill development but also affect the quality of clinical decision-making training. To address these issues, the Metaverse—a comprehensive platform that integrates VR, AR, and digital assets—presents unprecedented opportunities. The success of Metaverse applications in industries such as gaming and social networking has demonstrated its capacity to create highly realistic, interactive, and engaging virtual environments. By transferring these capabilities to medical education, the Metaverse can provide a safe and risk-free platform where medical students can practice complex surgical procedures, analyze cases, and refine their clinical decision-making skills (5, 6). This paper aims to explore the application of the Metaverse in surgical clinical teaching, analyzing its potential to enhance teaching effectiveness, overcome traditional limitations, and foster global medical education collaboration. Additionally, it discusses the technical challenges and ethical concerns associated with its implementation. As Metaverse technology continues to evolve, it holds the promise of becoming a vital component in future medical education, driving a comprehensive transformation in surgical clinical training (7, 8).

## 2 Concept and characteristics of the metaverse

### 2.1 The concept of metaverse

The term “metaverse,” coined in Neal Stephenson’s 1992 novel Snow Crash (9), refers to a virtual world that merges with the real world, driven by advancements in VR technology (10). Recent interest in the metaverse has been fueled by Web3, a decentralized iteration of the internet (11). However, the term is often used by companies as a buzzword to exaggerate technological progress, raising concerns about privacy, addiction, and safety—issues seen in the social media and gaming sectors (12). Concrete implementations of the metaverse are already visible, with platforms like Second Life and Roblox demonstrating the potential for immersive, interactive digital worlds. These platforms offer a glimpse into the metaverse’s possibilities, allowing users to engage, create, and transact in virtual spaces. Technologically, the metaverse merges digital technologies like VR, AR, and blockchain, creating a parallel digital world alongside the physical one (13). Educationally, it extends online learning with dynamic environments, applicable to fields like medical education. Philosophically, it represents a virtual space that expands human activity beyond the physical world (14), while societally, it transcends traditional boundaries of time and space (15). In essence, the metaverse is a higher-order form of the internet, offering a platform for creation, transactions, social interaction, and entertainment.

### 2.2 Characteristics of the metaverse

The metaverse offers a novel digital environment with four core features that have significant potential for medical education. Immersive virtuality, enabled by VR and AR devices such as Oculus Quest^®^ and HTC Vive™, allows users to enter high-fidelity 3D spaces, enhancing presence and facilitating realistic clinical simulations, especially in surgical training (16). Openness and interoperability, supported by standardized protocols and APIs, enable seamless integration across platforms like Second Life and Roblox, fostering collaboration and the embedding of educational tools and simulations (17, 18). User-generated content empowers individuals to create and customize digital environments, which supports tailored medical simulations, including virtual anatomy labs and procedural rehearsals (19). Lastly, blockchain technology underpins secure ownership and trade of digital assets, such as NFTs, providing verifiable certification of skills and simulation outcomes in education (20). Collectively, these features—immersive VR/AR, interoperability, user-driven content, and blockchain-backed assets—create a robust framework for experiential, data-rich medical training in virtual settings.

## 3 The application of metaverse in surgical clinical teaching

**Figure 1:**
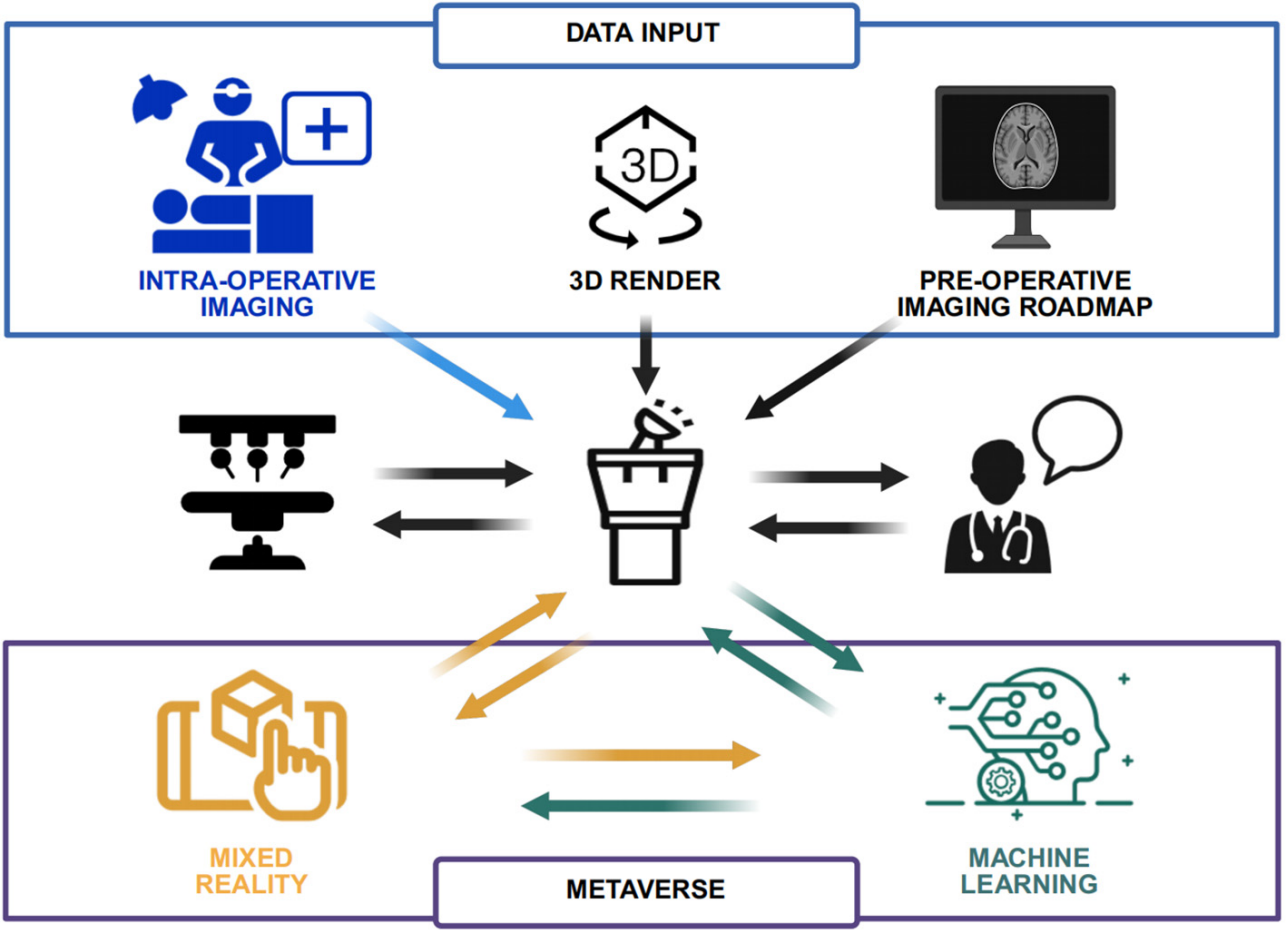
The robotic-surgical metaverse.

The emergence of the metaverse presents new possibilities for education by integrating real-world information resources with virtual teaching methods while also incorporating digital learning assets into traditional classroom settings (21). This two-way exchange creates a more realistic and comprehensive learning experience, effectively bridging gaps left unaddressed by conventional teaching models. In the metaverse, educators can employ virtual scenarios and interactive experiences to offer students immersive learning opportunities (**Figure 1**). For instance, in medical education, students can simulate real-life surgical procedures through VR technology, engaging in hands-on practice and collaborative teamwork. Preliminary studies at several medical schools have indicated that students who participated in VR-based surgical simulations demonstrated improvements in technical skills and clinical decision-making compared to those receiving traditional instruction (22). Moreover, the metaverse offers diverse virtual learning resources, such as virtual laboratories, historical recreations, and art exhibits, which broaden students’ horizons and enrich their learning experiences. By employing AR technology, educators can integrate virtual content into actual classroom instruction, enabling students to experience digital enhancements in a real-world context, thereby deepening their understanding and retention of complex concepts. Overall, the metaverse opens new avenues for educational development, addressing issues of uneven educational quality and digital isolation, and offering renewed support for high-quality, accessible education (23). **Table 1** showed the application of metaverse in surgical clinical teaching.

**Table 1:** The application of metaverse in surgical clinical teaching.

Technology	Role in Surgical Education	Examples in Surgical Training
Virtual Reality (VR)	Provides immersive, realistic 3D environments for practicing surgical procedures.	Oculus Quest, HTC Vive, Meta Quest 3 for VR simulations.
Augmented Reality (AR)	Enhances learning by integrating digital content with real-world clinical settings.	AR glasses and apps for anatomy visualization in the operating room.
Artificial Intelligence (AI)	Offers real-time feedback and personalized learning paths based on student performance.	AI-driven simulators that assess surgical skill levels and suggest improvements.

### 3.1 Exploring the application of metaverse in educational teaching scenarios

As an emerging technology, the metaverse is increasingly being explored in educational settings with the goal of improving learning outcomes and student engagement (24). Traditional teaching models often rely on teacher-centered approaches, where students passively receive information (25). In contrast, the metaverse fosters a more interactive and autonomous learning environment by allowing students to enter virtual worlds where they can engage in dialogues, complete interactive tasks, solve problems, and actively acquire knowledge and skills (26). The metaverse offers highly customizable and personalized learning experiences. By analyzing learning data and behavior patterns, the platform can tailor content to suit individual students’ needs, recommending materials that match their preferred learning styles and pace. Early experimental data from pilot programs at several universities suggest that such adaptive learning environments can enhance understanding and retention of complex subjects (27). Furthermore, this level of personalization extends to surgical education, where the metaverse enables students to engage in a variety of virtual surgical simulations, thus providing more opportunities for hands-on practice than traditional clinical settings allow. The application of the metaverse holds enormous potential in medical education, particularly in overcoming long-standing challenges such as limited practical opportunities and resource constraints. By providing highly simulated virtual clinical environments and rich digital learning resources, the metaverse enables students to safely engage in clinical operations and training. For example, several medical schools have implemented VR-based simulations where students can practice surgeries, diagnose virtual patients, and refine their clinical skills in a controlled, risk-free setting (28). A comprehensive meta-analysis by Mao et al. (27) systematically reviewed immersive virtual reality applications in surgical training, concluding that VR-based simulations consistently enhance technical proficiency and procedural accuracy across various surgical disciplines, with effect sizes comparable to or exceeding those of traditional training methods. McKnight et al. (7) further validated these findings, emphasizing that virtual reality and augmented reality effectively translate into improved surgical techniques by providing repeated, low-risk practice opportunities. In one pilot project, students who participated in virtual surgical simulations reported a 20% improvement in procedural confidence and a measurable enhancement in decision-making accuracy during subsequent real-world assessments (29). This aligns with recent findings from Neher et al. (28), who demonstrated that virtual reality-based assessments in medical education correlate strongly with improved performance in real clinical settings, particularly in domains requiring precise motor skills and rapid decision-making. The application of the metaverse in education revolves primarily around two core aspects: immersive learning environments and social interaction. Virtual classrooms can mimic real-world clinical settings by establishing rules and conditions that enhance realism. Within these environments, students can learn theoretical knowledge and perform practical experiments simultaneously, resulting in increased motivation and improved learning efficiency (30). Additionally, the adaptable nature of the metaverse facilitates the seamless interaction of diverse learning resources, enabling a fluid exchange of information between different virtual environments (31).

### 3.2 The current application status of metaverse in clinical teaching

The integration of the metaverse into clinical education is evolving, with encouraging pilot projects demonstrating its potential. Traditional medical training has been limited by a lack of practical opportunities and realistic clinical settings (32). The metaverse addresses these gaps by offering highly simulated virtual environments where students can practice clinical procedures. For instance, a study at a leading medical institution showed that students engaged in virtual diagnostic, therapeutic, and surgical procedures within a metaverse platform significantly improved their skills and decision-making abilities compared to peers in traditional settings (33). Additionally, virtual resources such as digital case libraries and anatomical models enable independent exploration of disease progression and pathology, enhancing students’ clinical knowledge retention (34).

One innovative approach within the metaverse is the use of first-person point-of-view (FPV) VR tutorials to teach medical procedures. This method employs 3D video filmed from the FPV perspective, offering a highly immersive experience for learners. In one study, medical learners were trained using an immersive VR application to perform lumbar punctures, an essential clinical skill. The IVRA-FPV group performed the procedure more efficiently with fewer errors and in less time compared to the traditional lecture group. This suggests that immersive VR-based tutorials can enhance procedural efficiency, allowing medical professionals to acquire techniques more reliably and in a shorter time, without the need for expensive physical simulators. This method could be a valuable addition to surgical education in the metaverse, further advancing the effectiveness of clinical training (35). Established platforms like Second Life have been integral to the development of virtual clinical teaching environments (36), and new technologies, such as intelligent robotic teachers and XR tools from platforms like VeyondMetaverse and Medicalholodeck, are further advancing surgical planning and training (37). These innovations address challenges such as limited hands-on practice opportunities, particularly in the wake of the pandemic (38).

Despite these promising advances, the application of the metaverse in clinical education is still in its preliminary stages and faces several challenges (39). High technological costs, significant equipment and network requirements, and difficulties in content development and maintenance remain major barriers (40). Potential solutions include pursuing government and institutional funding, developing low-cost alternatives, and investing in robust digital infrastructure. Additionally, ongoing research and iterative feedback from pilot programs are essential to optimize the effectiveness and reliability of metaverse-based clinical education (41). Future directions should focus on establishing standardized protocols, enhancing technological interoperability, and continuously assessing educational outcomes to ensure that these innovative platforms meet the rigorous demands of surgical training.

## 4 The advantages of metaverse in surgical clinical teaching

Metaverse technology provides extensive resources to address challenges in surgical clinical education, particularly those related to limited observation opportunities, insufficient practical training, and resource constraints in traditional clinical settings (42, 43). By constructing virtual scenarios based on real-life surgical situations, the metaverse teaching model seamlessly integrates theoretical knowledge with practical application (44). This approach enables the simulation of high-difficulty surgeries—procedures that are often inaccessible in actual clinical environments—by breaking them down into step-by-step virtual exercises. Through such simulations, students can observe and practice surgical operations repeatedly, deepening their understanding and equipping them to handle complex clinical problems in real situations (45, 46). Moreover, this technology offers additional benefits while also raising important considerations regarding psychological and ethical impacts, which will be discussed later (47).

### 4.1 Provide a highly simulated surgical environment

**Figure 2:**
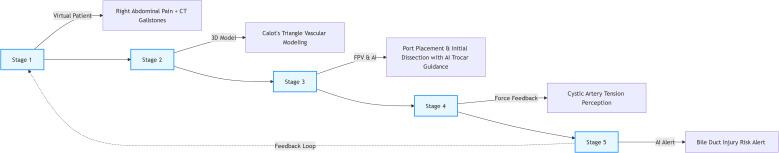
Progressive metaverse surgical training pathway for laparoscopic cholecystectomy.

Metaverse technology can deliver lifelike learning experiences that closely mimic real surgical scenarios through virtual environments featuring realistic visual and auditory effects (48). These environments are capable of simulating a variety of surgical procedures—including cardiac surgery and neurosurgery—allowing students to operate and observe within a safe, controlled setting. Using virtual surgical tools, instruments, and equipment, students can practice procedures and study anatomical structures, pathological sites, and surgical steps in detail (49). Additionally, virtual patients in these environments can simulate physiological responses and pain sensations akin to real patients, thereby helping students better understand the potential impacts of surgical interventions (50). From a technical standpoint, Unreal Engine 5’s Nanite and Lumen technologies enable sub-millimeter tissue modeling and dynamic lighting within the virtual environment (51). This high level of detail ensures that the visual complexity of tissues, organs, and surgical instruments closely mirrors real-world conditions. To simulate soft-tissue deformation and blood fluid dynamics, the system integrates NVIDIA PhysX, which provides robust physics-based interactions (52–54). Moreover, multi-user collaboration is facilitated, allowing up to six users to simultaneously perform or observe surgeries with minimal latency (55–57). These technological implementations jointly create a more realistic, immersive, and interactive virtual surgical environment, thereby enhancing the educational value of metaverse-based training. **Figure 2** showed the technological framework of Metaverse-enabled surgical training.

### 4.2 Personalized learning experience and feedback

The metaverse platform supports personalized learning by adapting to each student’s progress and individual needs (58). Intelligent systems can analyze a student’s performance, knowledge level, and skill mastery to recommend appropriate learning materials and exercises (59). In turn, the system offers timely feedback based on the students’ actions and decision outcomes, assisting them in identifying areas for improvement and supplementing their knowledge. This targeted feedback loop not only enhances learning efficiency but also helps students refine their operational skills and decision-making processes.

### 4.3 Interactive learning experience

Metaverse technology facilitates real-time interaction with virtual objects via VR devices and hand controllers (60). For example, students can use these controllers to perform simulated surgical procedures, observe clinical cases, and communicate with team members within the virtual environment. The dynamic responsiveness of virtual objects to students’ operations and commands enhances both the authenticity and engagement of the learning process. Furthermore, by working collaboratively in virtual teams to complete surgical tasks, students develop essential teamwork, collaboration, and communication skills (61).

### 4.4 Global interactive teaching and collaboration

By overcoming geographical barriers, metaverse technology promotes interactive and collaborative medical education on a global scale (62). Students can engage in virtual exchanges and collaborate with medical schools and educational institutions from diverse regions. Participation in virtual surgical operations, case discussions, and shared learning experiences offers access to a wide range of perspectives and resources. This globalized approach not only enriches learning but also fosters cross-cultural communication, understanding, and the development of international collaborative skills (63).

### 4.5 Teaching effectiveness evaluation and data analysis

Metaverse technology enables the real-time monitoring of students’ learning behaviors and performance through the integration of artificial intelligence and big data analytics (64). By analyzing operational records, decision pathways, and problem-solving processes, the system can assess students’ knowledge levels, skill mastery, and clinical reasoning. Additionally, by tracking learning behaviors and trajectories, the platform can identify individual learning preferences and challenges. This data-driven approach allows instructors to adjust teaching strategies and content promptly, ensuring that educational interventions are effectively tailored to enhance student outcomes (65).

To further enhance the evaluation process, a multi-dimensional evaluation matrix can be developed to assess teaching effectiveness across multiple dimensions, such as cognitive performance, technical proficiency, clinical decision-making, and interpersonal communication. This matrix would allow for a holistic assessment of each student’s progress, offering a more comprehensive view of their development in both technical and non-technical skills. Furthermore, an automated scoring algorithm can be integrated into the system to provide real-time feedback on student performance. This algorithm would process the data collected from student interactions in the metaverse environment and generate scores based on predefined criteria such as task completion time, accuracy, and the quality of decision-making. By incorporating machine learning models, the algorithm can adapt to individual students’ learning patterns, offering personalized evaluations and continuous adjustments to the scoring rubric. This automated system would reduce the subjectivity of traditional grading methods and provide more consistent and objective assessments. The combination of a multi-dimensional evaluation matrix and an automated scoring algorithm ensures that both quantitative and qualitative aspects of student learning are thoroughly assessed, leading to more precise and actionable insights for instructors. This approach would not only enhance the learning experience but also promote more effective, personalized teaching strategies.

### 4.6 Cross-disciplinary collaboration and knowledge integration

The metaverse facilitates interdisciplinary collaboration by enabling knowledge exchange between the medical field and other disciplines such as computer science, engineering, and psychology (66). For instance, computer science experts can enhance the realism and functionality of the virtual platforms, while engineers can develop and refine virtual surgical devices and instruments. Psychologists can contribute by analyzing the impact of virtual environments on learning behavior and cognitive processing. This cross-disciplinary collaboration fosters a comprehensive support system for medical education, enhancing students’ innovative thinking and overall competencies (67).

### 4.7 Virtual internships and remote teaching support

Metaverse technology also extends to virtual internships and remote teaching, offering solutions for geographical and resource-based limitations. Through virtual internships, students can experience simulated clinical scenarios, engage in practical operations, and make critical decisions in real-time under mentor guidance (68). For those unable to be physically present in clinical settings, remote teaching support via the metaverse provides an experience closely resembling in-person training. This model helps mitigate disparities in educational resources and ensures that high-quality surgical training is accessible to a broader range of students (69).

### 4.8 Psychological and ethical implications

While the advantages of metaverse technology in surgical clinical teaching are significant, it is important to address potential challenges related to its psychological and ethical impacts. Prolonged use of VR devices may lead to physical fatigue, eye strain, or discomfort, which can affect both students and educators (70). Strategies such as incorporating regular breaks, ergonomic VR equipment, and user-friendly interfaces are essential to mitigate these issues. Moreover, the virtual nature of the metaverse raises important ethical questions, particularly concerning data privacy, security, and behavioral norms within virtual environments (71). Institutions must develop robust data protection policies to ensure the confidentiality and security of student and patient information. In addition, clear guidelines and behavioral standards should be established to maintain professionalism and ethical conduct in virtual interactions. To further enhance data management strategies and behavioral oversight within metaverse-based teaching, adopting a federated learning approach ensures that students’ operation data is processed locally on their devices, with only anonymized metadata being uploaded to central servers, thereby protecting personal information and reducing the risk of data breaches (72). Meanwhile, leveraging smart contracts can automate the auditing of user actions in the virtual environment, for instance by restricting the improper use of instruments or inappropriate actions during simulated procedures. These self-executing contracts can detect deviations in real time and issue alerts or corrective measures. Addressing these challenges is critical to ensuring that the integration of metaverse technology in surgical education is both effective and responsible. By leveraging the immersive capabilities of the metaverse while proactively addressing its challenges, surgical clinical teaching can be transformed into a more engaging, accessible, and effective educational experience (73).

### 4.9 Limitations and practical constraints

While the metaverse offers transformative potential in surgical education, its limitations warrant balanced consideration to avoid overstated enthusiasm. Technically, current haptic feedback systems (e.g., HaptX Gloves) struggle to replicate the nuanced texture differences between tissues—for example, distinguishing the friability of cirrhotic liver from normal hepatic parenchyma or the elasticity of intestinal mucosa versus pancreatic tissue (52, 54). This discrepancy may lead to misalignment between virtual skill acquisition and real surgical tactile perception. Over-reliance on virtual simulations also poses risks: virtual environments, by design, simplify complex clinical variables (e.g., unexpected intraoperative bleeding, equipment malfunctions) that are critical in real-world scenarios (47). Trainees overexposed to standardized virtual cases may develop deficits in adaptive decision-making, a core competency in surgical practice. Cost remains a substantial barrier. High-fidelity VR headsets (e.g., Varjo XR-4) and multi-user simulation platforms require initial investments exceeding $50,000 per setup, with annual maintenance costs adding 15–20% (40, 43). This creates a resource disparity, where only well-funded institutions can adopt such technologies, potentially widening educational gaps between large and small medical schools.

## 5 Outlook of Metaverse in medical clinical teaching

The emerging concept of the metaverse has the potential to revolutionize surgical clinical education by addressing limitations inherent in traditional teaching methods (62). The immersive experience provided by metaverse-based education not only enables students to gain a comprehensive understanding of clinical phenomena but also stimulates creative thinking, fostering novel ideas and innovations. As the ultimate form of visual immersion, the metaverse integrates various digital technologies that embody human externalized intelligence, often surpassing the cognitive scope of conventional mathematical reasoning and scientific experimentation (74). This integration positions the metaverse as a fundamental approach to understanding natural laws and reshaping online education in the era of intelligent learning. However, current perspectives and future visions of the metaverse remain highly forward-looking, with existing technologies and research designs still in their early, often simplistic, stages. With these limitations in mind, the following considerations and suggestions are proposed for the further development of metaverse-based clinical teaching.

### 5.1 Establishing a comprehensive plan for metaverse clinical teaching

At present, there is no systematic plan for the application of the metaverse in medical education, as existing policy documents primarily focus on VR applications without addressing the specific needs of various clinical disciplines. Learning materials and teaching methods remain fragmented, lacking unified organization, comprehensive evaluation, and iterative feedback mechanisms. To move forward, educational authorities and institutions must strengthen top-level design and implement a dedicated plan for integrating the metaverse into clinical teaching. This should include selecting leading institutions to pioneer pilot projects that provide exemplary cases detailing necessary teaching content, faculty expertise, and resource allocation, as well as establishing robust evaluation and feedback mechanisms to identify and rectify deficiencies during implementation. Furthermore, it is crucial to explore the synergy between the metaverse and emerging technologies such as AI and big data analytics, which can offer predictive insights, adaptive learning pathways, and advanced simulation capabilities, thereby further enhancing the teaching effectiveness and personalization of clinical education. Additionally, collecting diverse user feedback from teachers and students across different disciplines is key to understanding varied needs and developing a broad market of tailored online metaverse products.

To streamline and standardize the metaverse-based clinical teaching process, a five-stage progressive teaching framework—”Patient Evaluation → Anatomy/Imaging Learning → Mimetic Simulation → Hands-on Practice → Feedback & Correction”—is proposed, building on and expanding the original three-tiered model (74, 75). Stage 1: VR-Based Patient Evaluation and Diagnosis, as the foundational stage, immerses students in virtual clinical encounters, simulating the full spectrum of patient assessment; leveraging patient-specific digital twins reconstructed from real clinical data (e.g., medical histories, symptom presentations, and preliminary test results), students practice history-taking, virtual physical examinations (e.g., palpating abdominal masses via haptic feedback gloves with tactile response simulation), and interpreting baseline diagnostic metrics, with advanced modules integrating virtual imaging data (CT/MRI scans) into the 3D patient model to enable students to correlate clinical findings with radiological features—strengthening the critical link between diagnosis and surgical planning, thereby cultivating clinical reasoning skills essential for translating patient needs into intervention strategies (34, 68). Following this, Stage 2: 3D Anatomy and Imaging Learning, building on diagnostic insights, focuses on high-precision anatomical exploration, using submillimeter 3D models reconstructed from patient CT/MRI data (powered by Unreal Engine 5 Nanite) for students to interact with dynamic anatomical structures, including pathological variations such as vascular anomalies or tissue adhesions, while NVIDIA PhysX-driven simulations visualize how disease processes (e.g., gallstone impaction causing tissue inflammation) alter anatomical relationships, reinforcing the connection between pathology and surgical approach to ensure mastery of structural context before procedural practice (51, 74). Next, Stage 3: FPV Simulation Training (Mimetic Stage) integrates first-person point-of-view (FPV) VR tutorials (35) to emphasize observational learning and mimetic practice—addressing the critical step between knowledge acquisition and hands-on performance—where students engage with 3D FPV recordings of expert-led procedures (e.g., laparoscopic cholecystectomy), experience the surgical field from the operator’s perspective, and replicate key steps (e.g., instrument handling, tissue dissection sequences) in a low-risk virtual environment guided by AI prompts, focusing on spatial awareness and motor planning without haptic feedback, aligning with the principle that “observation and experiential learning precede reproduction” to lay the groundwork for procedural proficiency (35, 73). Subsequently, Stage 4: Multimodal Surgical Simulation (Hands-on Practice) advances to immersive hands-on training with haptic feedback (e.g., HaptX Gloves), enabling students to perform full surgical procedures on virtual patients, with simulations incorporating realistic tissue resistance (e.g., 0.8–1.2N force feedback for gallbladder grasping) and dynamic clinical events (e.g., unexpected intraoperative bleeding or equipment malfunctions) to bridge the gap between mimetic practice and real-world complexity, while multi-user collaboration features allow students to work with virtual surgical teams (e.g., communicating with virtual nurses for instrument passes) to enhance teamwork skills (52, 54). Finally, Stage 5: AI-Driven Real-Time Feedback and Correction involves an AI system leveraging natural language processing to analyze students’ operation logs and behavioral data from the virtual environment, providing instant error alerts (e.g., “Electrocautery hook <2mm from common hepatic duct”) and personalized improvement suggestions (e.g., “Adjust grasp force to reduce tissue trauma”), with blockchain technology (Ethereum sidechain) securely documenting each operational step and feedback record as immutable hashes to ensure data integrity, track learning trajectories, and facilitate inter-institutional data sharing and progression verification (76). This five-stage framework is designed to integrate seamlessly with traditional teaching methods, forming a “virtual-real hybrid” ecosystem where preoperative foundational knowledge from textbooks and physical models is reinforced in Stages 1 and 2; virtual practice in Stages 3 and 4 is complemented by traditional animal lab sessions to refine tactile perception; and assessment combines metaverse-generated metrics (e.g., procedural accuracy) with evaluator assessments of clinical judgment.

### 5.2 Emphasizing ethical issues in the development of metaverse clinical teaching

Although current research predominantly highlights the positive effects of metaverse-based education on classroom engagement and teaching effectiveness, ethical concerns are gaining attention as the metaverse becomes more integrated into clinical teaching. It is imperative to address data privacy and security by prioritizing the protection of teachers’ and students’ information; institutions must develop and enforce robust data protection policies to manage and secure sensitive feedbacks. The five-stage progressive teaching framework of Metaverse-enabled surgical trainingck data without compromising personal information. Additionally, cultivating appropriate ethical values within the metaverse is essential given the diverse cultural backgrounds of learners, so educators should guide students in developing a balanced worldview and proper conduct in virtual environments. Furthermore, the immersive nature of the metaverse can lead to risks such as addiction or cognitive overload, making it necessary to implement strategies like scheduled breaks, monitoring usage patterns, and integrating wellness checks, while also helping students manage their virtual selves and avoid over-reliance on digital interfaces. Finally, reflecting on the long-term impact of metaverse-based education, it is crucial to consider how these immersive experiences will shape the professional development of future clinicians by evaluating how virtual practice environments contribute to skill retention, adaptability, and decision-making in real-world clinical settings.

### 5.3 Establishing a comprehensive teaching theory system

Current intelligent technologies on campuses are largely limited to demonstrations and basic communication, with software development being predominantly technology-driven and lacking robust theoretical support for clinical teaching innovations. To fully harness the potential of the Metaverse, it is necessary to develop new educational theories by drawing upon broader educational research to establish models centered on embodied cognition tailored for Metaverse-based medical clinical learning. This should include the innovative design of teaching content that leverages the unique features of the Metaverse, with cognitive science theories forming a critical foundation. Sweller’s Cognitive Load Theory, a cornerstone of effective VR-based learning, emphasizes that optimal learning occurs when cognitive load—the mental effort required to process information—is carefully managed (7, 27). In Metaverse surgical education, this translates to designing virtual environments that minimize extraneous cognitive load (e.g., excessive non-essential visual details) while directing learners’ attention to core clinical concepts and procedural steps. For instance, structuring virtual surgical scenarios to prioritize critical decision points (e.g., identifying the cystic duct during cholecystectomy) over redundant background information ensures cognitive resources are focused on mastering essential skills. Building on this foundation, Neuwirth and Ros (35) highlighted a critical risk in immersive VR: while the technology enables hyper-detailed content presentation, unregulated information overload can paradoxically hinder learning. Their analysis of surgical training modules demonstrated that virtual scenarios overloaded with excessive anatomical textures, instrument labels, or concurrent procedural prompts impede knowledge retention, as learners struggle to filter critical information from noise. To address this, we propose a “cognitive load optimization principle” for Metaverse curriculum design: AI algorithms dynamically adjust information intensity based on learner proficiency (e.g., simplifying non-critical tissue details for novices, gradually increasing complexity for advanced trainees) and implement “hierarchical information display” (prioritizing real-time operation prompts over secondary anatomical annotations). This ensures technical fidelity serves, rather than overrides, educational goals. Complementing these insights, Follower (35, 49) provided a timeless framework for aligning VR technology with pedagogical goals, even in pre-immersive VR contexts. His “pedagogy-technology adaptation model” emphasizes that VR effectiveness depends on the tripartite alignment of teaching objectives, technical capabilities, and learners’ cognitive characteristics. For example, foundational skills (e.g., suturing) benefit from simplified virtual environments to reinforce motor memory, while complex decision-making training (e.g., managing intraoperative bleeding) requires strategic increases in scenario complexity to challenge clinical reasoning. Extending this to the Metaverse, we introduce a “dynamic adaptive pedagogy”: AI analyzes learner performance data (e.g., error patterns, response times) to real-time adjust virtual scenario complexity, information density, and feedback modes. This ensures technical features (e.g., haptic feedback, multi-user collaboration) are calibrated to specific learning goals, avoiding technological spectacle at the expense of educational value.

### 5.4 Institutional challenges and mitigation strategies

The integration of metaverse technology into surgical education faces significant institutional barriers, including academic inertia within traditional medical institutions where clinical excellence has long been prioritized over technological innovation—senior faculty, often trained in lecture-based and operative video-driven pedagogy, may resist adopting virtual simulations, viewing them as supplementary rather than integral to core curricula, compounded by a lack of standardized training for educators in metaverse tools that creates a skills gap hindering implementation (32, 38); concurrent with this inertia is the growing influence of private commercial entities in medical education, as platforms like Osso VR, which offer pre-packaged, standardized metaverse training modules with user-friendly interfaces and scalable content, rapidly capture market share (43), yet risk sidelining public universities and smaller institutions unable to afford licensing fees (often exceeding $20,000 annually per program) or high-end hardware, widening existing disparities by concentrating advanced training resources in well-funded institutions and marginalizing those with limited budgets (40, 41). To address these challenges, multi-faceted mitigation strategies are proposed: public-private alliances, where large academic centers with established metaverse infrastructure partner with smaller institutions to share access to virtual platforms (e.g., a regional consortium centralizing server hosting and content development to allow members access at fractional costs) (62); government and institutional subsidies, such as targeted funding programs covering 50% of hardware costs (e.g., VR headsets, haptic gloves) to lower entry barriers for resource-constrained schools and subsidizing faculty training workshops on metaverse pedagogy to address skill gaps and foster buy-in (39); and curriculum and evaluation reform, with medical schools revising promotion and tenure criteria to explicitly include virtual teaching competence (incentivizing faculty adoption) and accrediting bodies mandating metaverse integration in core surgical curricula to ensure standardized adoption across institutions (28, 33).

## 6 Conclusion

The integration of the Metaverse into surgical education marks a transformative advancement in medical training, offering immersive environments and personalized learning pathways. It not only improves practical skills and clinical decision-making but also fosters global collaboration. While existing VR and simulation-based training have shown promise, the Metaverse expands on these by incorporating 3D anatomical models, real-time surgical simulations, and AI-driven feedback, which offers a more holistic and scalable learning experience. However, challenges such as high technological costs, equipment requirements, and ethical issues remain. Unlike traditional methods, the Metaverse’s immersive nature presents new challenges like cognitive overload, which must be addressed. To advance, we propose an original theoretical model for Metaverse-based surgical education, combining Adaptive Immersion, Personalized Learning Pathways, and AI-Augmented Competency Assessment. This model dynamically adjusts content based on learner progress and emotional state, offering tailored pathways and real-time AI-driven feedback to enhance performance across multiple dimensions, including accuracy, decision-making, and ethical judgment. By addressing these challenges and implementing this model, the Metaverse can shape a new generation of healthcare professionals who are more skilled, adaptable, and globally connected.
